# The First Year of the Psychiatric Diseases Section

**DOI:** 10.3390/brainsci13010041

**Published:** 2022-12-24

**Authors:** Robert E. Kelly

**Affiliations:** 1 Department of Psychiatry, Weill Cornell Medicine, White Plains, NY 10605, USA; rek2005@med.cornell.edu or robert.kelly@med.lu.se; 2 Department of Clinical Sciences, Lund University, 22100 Lund, Sweden

Since its inception in May 2021, the Psychiatric Diseases Section of *Brain Sciences* has grown to include a staff of 28 academic editors with expertise related to clinical psychiatry, in addition to its supporting staff of managing and English-language editors. Our mission is to gather “the best available scientific evidence to guide clinical practice in the treatment of psychiatric illnesses”. With this goal in mind, an average of approximately 10 articles per month are currently published within the Section, last year totaling 56 articles. Here are examples of our most popular articles from 2021.

Acevedo et al. [1] provide a comprehensive systematic review of treatment of OCD and related disorders (OCRD, including body dysmorphic disorder, trichotillomania, excoriation disorder, and hoarding disorder; and including Tourette syndrome, given its high comorbidity with OCD) using therapeutic neurostimulation (transcranial magnetic stimulation, transcranial direct current stimulation, deep brain stimulation, and electroconvulsive therapy). The authors succeed in their aims of “(1) reviewing the clinical significance of neurostimulation therapies for OCD and OCRD; (2) making clinical recommendations for optimal stimulation protocols; (3) reporting on limitations and strengths of current neurostimulation protocols in order to (4) identify future directions for greater consistency and validity of treatment effects”. Despite concise presentation, 81 pages were needed for this article, including 250 references, to provide an up-to-date reference work that covers the broad and rapidly changing subject of treatment of OCRD with neurostimulation.

Martinotti et al. [2] also provide a review of treatment of OCD, using ketamine and esketamine. In contrast with Acevedo et al., they broaden their diagnostic focus to include substance use disorders (SUD) and eating disorders (ED), which they consider related to OCD because “all involve recurring and intrusive thoughts and generate associated compulsive behavior”. The authors note that advantages of ketamine or esketamine over other medical treatments of OCD, SUD, or ED include “no requirement for daily administration”; “in the case of concomitant medications, the possibility to reduce doses”; and “the availability of nasal spray formulations … in the case of esketamine”. The authors find some results encouraging and supporting the possibility of effective treatment for these disorders with ketamine or esketamine, but conclude “that results are still preliminary, and mechanisms are incompletely understood”.

Salazar de Pablo et al. [3] provide a systematic review and meta-analysis on the prevalence of adolescents and young adults with a clinical high-risk for psychosis (CHR-P), to facilitate the early detection of such individuals, with the goal of improving the course of psychotic disorders. The authors estimate that the prevalence of stringently defined CHR-P (not merely having any psychotic-like experiences) among young people is 1.7%, and that 20% or more of them would later develop a psychotic disorder. They recommend the use of extensive CHR-P assessments for clinical samples, but not for the general population, unless “adequate risk enrichment strategies can be implemented via pre-screening assessments”.

The COVID-19 pandemic’s impact on mental health has been a major subject of study. Menculini et al. [4] summarize this impact from societal, psychological, and biological perspectives, before reporting their findings from the COVID Mental Health Trial (COMET), “a multicentric collaborative study carried out in Italy, one of the Western countries most severely hit by the pandemic”. Their aims were to “evaluate the use of mental health resources during the first pandemic wave among the Italian general population” and to “identify factors associated with access to mental health services”. They investigated the use of mental health resources, finding associations between referrals to mental health resources during the pandemic and both the environmental factors and psychopathological features of the people referred. Sampogna et al. [5] also use data from the COMET study to “describe the levels of coping strategies and resilience in the Italian general population during the first wave of the pandemic” and to “evaluate the protective role of coping strategies and resilience on the levels of depressive, anxiety and stress symptoms”. The theme of coping strategies is further studied in Romanian patients with COVID-19 by Dehelean et al. [6] who explore the relationships among COVID disease severity, psychiatric response, and coping strategies utilized. Finally, Gebska et al. [7] explore the relationships among symptoms of temporomandibular disorders, type D personality, and depression among Polish physiotherapy students during the COVID-19 pandemic.

These are a few selected articles from our first year in the Psychiatric Diseases Section. Our success continues this year, with nearly twice as many articles expected by year-end 2022, compared with 2021. We look forward to our next report on these works, next year.

## Data Availability

No data were used for this article.
